# Studying Acute Coronary Syndrome Through the World Wide Web: Experiences and Lessons

**DOI:** 10.2196/resprot.6788

**Published:** 2017-10-13

**Authors:** Angelo A Alonzo

**Affiliations:** ^1^ Department of Sociology and School of Nursing Ohio State University and Yale University Columbus, OH United States

**Keywords:** acute coronary syndrome, care-seeking, Internet study, Internet recruitment

## Abstract

This study details my viewpoint on the experiences, lessons, and assessments of conducting a national study on care-seeking behavior for heart attack in the United States utilizing the World Wide Web. The Yale Heart Study (YHS) was funded by the National Heart, Lung, and Blood Institute (NHLBI) of the National Institutes of Health (NIH). Grounded on two prior studies, the YHS combined a Web-based interview survey instrument; ads placed on the Internet; flyers and posters in public libraries, senior centers, and rehabilitation centers; information on chat rooms; a viral marketing strategy; and print ads to attract potential participants to share their heart attack experiences. Along the way, the grant was transferred from Ohio State University (OSU) to Yale University, and significant administrative, information technology, and personnel challenges ensued that materially delayed the study’s execution. Overall, the use of the Internet to collect data on care-seeking behavior is very time consuming and emergent. The cost of using the Web was approximately 31% less expensive than that of face-to-face interviews. However, the quality of the data may have suffered because of the absence of some data compared with interviewing participants. Yet the representativeness of the 1154 usable surveys appears good, with the exception of a dearth of African American participants.

## Introduction

Several years ago, the idea of conducting a study on delay in seeking care for heart attack on the World Wide Web came to me when I began receiving invitations to complete various Web-based surveys. I had, at the time, been studying care-seeking behavior with regard to heart attack or acute coronary syndrome (ACS) for almost four decades. The goal in studying ACS delay is to improve health outcome by understanding why and how people get care during an ACS event where basically *time is muscle*; the longer it takes to get care, the less heart muscle survives, and more disability ensues. In the first hour, the risk of cardiac death is the highest. At present, delayed care-seeking behavior prevents individuals from obtaining the full therapeutic benefit of hospital-based reperfusion therapy that reduces the morbid and mortal consequences of an ACS.

In this study, I provide my viewpoint on the details of, and my perseverations on, my travails and triumphs and the lessons and liabilities of my journey in ultimately collecting 1154 usable Web-based interview surveys for analysis from 134,421 clicks to the study website and the 279,834,651 million ads displayed on social media and the unknown number of Yale Heart Study (YHS) flyers posted across the United States in public libraries, senior citizen centers, and cardiac rehabilitation centers. Although my viewpoint does reflect all the experiences common to conducting a Web-based behavioral study, I did experience a broad range of contingencies associated with conducting this study that may be useful to persons contemplating a Web-based study.

## Study Description, Aims, and Background

The primary aim of the study I proposed was to increase our understanding of care-seeking behavior surrounding ACS by using an integrated self-regulatory care-seeking model (ISCM). The innovative part of this study was using a World Wide Web–based ISCM survey instrument. Prior studies on delays in seeking care for ACS generally rely on measures of time duration from acute symptom onset (ASO) to hospital emergency department (ED) arrival and measures of demographic and clinical variables extracted from emergency medical system (EMS) logs or ED charts and, at times, ACS registries. By relying on such limited data sources, these studies infrequently take into consideration the complexity of the social and behavioral processes by which individuals and their families and others make decisions to seek care for ACS symptoms. This study sought to extend my work and that of my colleagues from two communities: Silver Spring, Maryland and Columbus, Ohio, described below. I thought that by using the Web, we could overcome the limitations of one hospital, EMS, or community to obtain a more representative sample of ACS care-seeking experiences and to test the ISCM. I also considered additional influential factors in seeking care for ACS, for example, were ACS symptoms what the individual expected a heart attack to be like? [1] or how does cumulative adversity [2] and post-traumatic stress disorder (PTSD), if present, influence care-seeking behavior [3]?

What would eventually become the YHS was funded in a traditional manner by the National Heart, Lung, and Blood Institute (NHLBI) of the National Institutes of Health (NIH). I applied for and received an RO1 level grant that was to run for 3 years with the aim of applying the ISCM to ACS care-seeking behavior and to delineate decision points and circumstances critical to producing care-seeking patterns that are efficient and expeditious or are protracted and delayed. The care-seeking process in the ISCM was viewed in terms of five analytic care-seeking phases from warning or premonitory symptoms to ED arrival. By noting the presence or absence of three central phases, the self, lay, and medical evaluation phases, it would be possible to distinguish care-seeking patterns and to record the duration of each care-seeking phase and the total duration of each care-seeking path to the ED. The ISCM fundamentally assumes that coping behaviors emerge over time and that initially care-seeking behaviors are constrained by demographic and structural influences. However, as the self-regulatory [4] and coping processes [5] emerge over the course of the ACS event, the influence of structural influences diminishes, and emergent ACS symptom evaluations, advice from others and health care providers, and emerging symptoms and emotions come to dominate the care-seeking process and directly predict the duration of ACS care-seeking. The goal was to capture the processes by which the study participants found their way to the ED and to delineate conditional care-seeking models and their predictors.

In the two earlier studies, interviews were conducted with hospitalized ACS patients either by myself or a physician—we were both US public health officers assigned to the NIH at the time—at a single hospital in Bethesda, or by a cadre of 6 “nurse epidemiologists” who interviewed patients hospitalized in one of 6 hospitals in Columbus. For the YHS, I took the interview schedules used in the two prior studies and extended the emphasis on emergent qualities of the ACS event noted above. The ISCM interview schedule was to be hosted on the Web, and potential participants who had experienced an ACS event would be asked to come to the YHS website to complete the YHS interview survey.

Having interviewed patients in the Bethesda study and supervised and reviewed interviews completed in Columbus, I thought we could successfully bring the YHS interview to the Web, and it could be easily found, downloaded, navigated, and completed. I also thought deep down that we would optimistically obtain a minimum of 10,000 study participants, even though we had calculated that a sample of only 2314 YHS participants was needed to test the ISMC model. I was dreadfully wrong, as will be made clear below. A warning: Doing a Web-based study is difficult and very time consuming, however, not as time consuming as doing face-to-face interviews. Whereas the intensity of the face-to-face interview comes from locating, asking permission, and interviewing potential participants, a Web-based study presents daily challenges centering on clicks to the study website, how many clicks to the site does it takes before someone signs up to participate, and whether that participant is going to complete the survey.

## Research at the Ohio State University

### The Survey Instrument

Over the years I had learned that to successfully win funding for research, you needed to complete some of the work for which you are requesting funding. With this in mind, and my prior work, I began to build the necessary survey instrument and to pilot-test it as a way of demonstrating the feasibility of a Web-based study of ACS events.

The YHS survey instrument, as noted, was derived from the two prior studies. Piloting the initial self-administered YHS survey instrument was accomplished using a paper-and-pencil version of the survey with 13 study participants who volunteered through an ad run in the Ohio State University (OSU) electronic staff newsletter. Volunteers sent me their name and address, and I sent them the questionnaire and a postage-paid return envelope. Interestingly enough, none of the volunteers who requested the questionnaire were the subjects, but they were going to give it to a family member or “friend” who was the potential participant. Although I had their postal addresses, all interviews were returned anonymously.

From the Bethesda study, it was obvious that there were patterns to ACS care-seeking behavior; not everyone reached the ED at the same time and used the same resources. Whereas an interviewer can skip nonrelevant questions, I also had to design a survey that could be self-administered and only asked relevant questions so as not to overburden participants. Therefore, I was extremely interested in the skip patterns presented in the paper-and-pencil format, which were the heart of a “self-tailoring” survey, and whether unique paths through the survey could be easily followed. The skip patterns, or algorithms, made sense to pilot participants and became the basis of the self-tailored YHS survey instrument; for example, if the participants indicated that they did not consult a health care provider before ED arrival, a part of the survey covering prehospital provider contact was skipped over. To facilitate readability, a technical writing specialist had the survey read at the 5th to 6th grade level. Also, for terms that might need definition, participants only needed to hover their cursor over the term, and a pop-up balloon with a definition would appear derived from lay literature on health information derived from the National Library of Medicine or NHLBI websites where health terms are defined for lay audiences [6,7].

With this tested survey instrument in hand, I next looked for a means to bring it to the Web. I needed a Web-based, self-administered survey that had an efficient common gateway interface that allowed a potential study participant’s computer to download and upload information quickly to facilitate survey progression, that was sensitive to a participant’s connection speed, that allowed for participants to easily modify font size, and that required minimal computer skills.

### Information Technology

I was basically a consumer of information technology (IT) relying on IT personnel and software programs with a user-friendly interface. So, I knew that I was not going to build the YHS survey instrument or the YHS website. I had a good working relationship with the IT staff in my department, and yet, the IT subculture surprised me from my initial approaches to having the YHS survey instrument designed by students in IT classes at the OSU to incompatibility issues not only between software and servers but also between the website designer and OSU IT graduate students maintaining the YHS website on a Yale server. Traversing the IT landscape at both OSU and Yale was instructive on many levels, yet, lessons learned came at a great price in terms of delaying the study’s execution.

Shortly after piloting the survey instrument, I saw an email solicitation from an OSU IT faculty member needing faculty projects to work on for her class in system design architecture. Having no funding to produce a Web-based version of the survey, I wrote to her with my needs; she accepted me as her class project for the quarter. I was their class client; her class had five groups of 4 students, and each designed their version of the required system. I attended three classes; the first to tell the students what I thought I needed, with students asking me questions to obtain information they needed, and what decisions I needed to make for them to be efficient in their design. The next session was a progress report of what each group had produced to solve the design problems and, again, focused on more decisions to be made. At the final session, each group presented their final system design solution. The faculty member assessed which design was the best and that is what I carried away.

I then took this system design to another IT class in which my project was one of several being worked on by student groups. In this class, the actual code for YHS survey was written, and the instructor for that class became the IT consultant for the eventual YHS research proposal. Of the students who worked on the second stage, only one wished to continue with the project to the point of completing a final survey that could be pilot-tested to demonstrate the feasibility of collecting complex behavioral data using a Web-based survey instrument.

The student who volunteered to continue working on the study indicated that he would like to do this work in his spare time because he needed to work over the summer to support himself; I doubted this arrangement. Fortunately, OSU IT ran a summer intern program to facilitate student work with real-world projects, again drawing from faculty and the public who needed Web development services. We applied for the summer intern program and were accepted. He would receive a summer stipend and participate in enhanced IT seminars, and I would receive a functional Web survey that could be piloted to demonstrate feasibility to the NIH.

The Web-based survey developed at OSU was not entirely completed as, what I call it, the “black box,” or data complier still needed to be built; the complier converted a click on the Web-based survey’s radio button options into data points to be taken into statistical analysis programs such as Statistical Analysis System (SAS; SAS Institute Inc, North Carolina) or Statistical Package for the Social Sciences (SPSS; IBM Corporation, New York). However, the survey was functional enough in terms of collecting responses, and the preliminary output allowed me to demonstrate to NIH how many subjects participated in the Web-based pilot study and their demographic characteristics. The results looked very good in terms of hosting the survey instrument on an OSU server, having a functional instrument, and collecting a respectable demographic distribution of participants.

### Pilot Study on Cape Cod

Where did our initial participants come from? For the initial survey pilot test I turned to Mended Hearts—a national heart attack self-help organization—to assist in locating members willing to share their ACS experiences. After considerable delay, a recurrent theme in this study, and administrative processing, the Mended Hearts national organization agreed to run a paragraph in their electronic newsletter, asking local chapters if they would consider running a solicitation notice in their local chapter newsletter. A chapter in the Cape Cod, Massachusetts (MA) area responded positively to our request, and many of the pilot participants came from that region. Whereas 27 pilot participants completed the Web-based survey, 18 partially completed the survey. This partial group, while not originally seen as prophetic, was, in fact, very telling as will be seen below.

The YHS server for the pilot study was housed in the Department of Sociology at OSU, my home department at the time. There was sufficient server space and personnel to support the website in the OSU environment. With pilot data collected and the grant proposal written and sent off, I waited to see the outcome. Lo and behold, several months later we were funded! At about the same time, my partner received an offer of a position at Yale University. Although it was actually my partner they wanted, because of the new grant, they would also take me as part of the package and provide a research scientist position, thus allowing me to complete the study.

## Research at Yale University

### Arriving at Yale

At Yale, and in particular the Yale School of Nursing (YSN), I would conduct the study and eventually begin to work on other projects, advise graduate students, assist faculty with grants, do guest lecturing, and, in general, participate in YSN’s research and teaching missions. However, there was one difference in the two environments that either represented a true cultural difference or my lack of credibility as a newcomer. Having a research grant in hand is a great feeling, but moving it forward is an entirely different matter. I found myself in a new academic environment where considerable work had to be done just to bring the study to Yale and much more to get it up and running. Perhaps because I was a newcomer, I frequently felt as if I was being told what I had done wrong rather than someone helping me do it correctly in the first place. While at OSU, I had been accustomed to support colleagues that listened to what I needed to accomplish, and provided guidance within OSU’s framework, offering resources and/or how to use a variance to accomplish a task.

### Information Technology Surprises

Despite the move to Yale, I wanted to leave the survey instrument on the OSU server because it is where it was developed, and people were invested in the study. However, the subculture of IT intervened, and the IT director at the Department of Sociology, OSU, did not want Yale IT to have access to the OSU server because as you will recall, the survey’s compiler was not complete and IT personnel from YSN were going to complete it as part of the transfer process. Therefore, I proposed transferring the entire study to Yale. This was not a bad idea but not a good one in practice. I was warned by my OSU grant officer that it was not a good thing to do, and it would take a long time to transfer the grant. Unfortunately, she was more than correct on both counts.

It took more than a year for the transfer to be completed. However, since I had no actual funds in hand when arriving at Yale, no one I needed services from wanted to invest much time in the study. More problematic were the changes in the expected support from the YSN IT unit. Furthermore, it was unbeknown to me at the time of our arrival at Yale that YSN was in the process of going from an autonomous IT unit to being folded into the larger Yale IT environment for economic and efficiency reasons. I was promised that YSN’s IT would complete the survey’s data complier, install it on YSN servers, and support it over the 2 years of data collection. However, the merging of YSN IT into the larger IT meant that I could not complete the survey instrument because Yale IT did not support the original PHP programming language used to build the survey. However, after the transition to central IT, PHP programs would probably be allowed to run in the larger IT environment. In the interim, however, no Yale IT personnel were allowed to write PHP code! Consequently, I would have to do one of two things, in addition to waiting—either have Yale IT rewrite the survey in compatible code or go outside of Yale to secure the services of a PHP programmer. However, I did have a third idea that I tried, which was going to the Yale’s Department of Computer Science and engaging a graduate student programmer. I was told by a placement administrator that this would not work, because the department was not oriented toward applied endeavors. Yet, one student did respond to an ad posted on their placement system; the student was only peripherally familiar with PHP and wanted to work on the data complier at home abroad over the summer. I did not find this option appealing.

Thus, I decided, fatefully, to go outside Yale as I had already put so much time and effort into getting the survey instrument designed and implemented. Additionally, the cost of Yale rewriting the survey seemed absorbent, and there was no room in the budget to cover the costs of such a rewrite. However, in retrospect I did have adequate funding to accomplish this rewrite, despite Yale’s overhead being greater than that of OSU’s—thus, another misjudgment on my part.

### Information Technology and Going Outside

Going outside the Yale IT environs proved very difficult. No one at Yale IT would officially, or unofficially, recommend a PHP programmer who could do the work; it was against Yale policy to recommend outside vendors. I finally tried the IT person at the Department of Sociology at Yale, who I thought should be familiar with surveys and survey hosting and thus might know of someone to assist me. I obtained a name, followed up, interviewed him, and we agreed on a price and a timeline. However, it took months to get his contract finalized with frequent conversation with contract specialists in Yale legal and the YSN business office. The programmer wanted partial payment up front, which was contrary to Yale contract policies. He said he had previously designed surveys, was doing contract work for a Yale department at the time, and he could have the project completed in 2 weeks’ time.

At the time of hire, he was very confident, but he did not know enough about data compliers, and I found myself answering very basic questions about how to handle strings of variables and comma separated variable files. Eventually after months of frustration and some progress, I had to let him go, pay his total fee because he had spent so much time on the project, and return the survey to OSU to be completed by IT graduate students, where I should have left it in the first place.

### How the Yale Heart Study (YHS) Site Worked and Where It Was Housed

When completed, the YHS survey instrument worked extremely well, it was stable, downloaded quickly, and collected data as designed. However, as mentioned above, the language it was written in was not compatible with Yale IT servers, and thus, it could not run on Yale central servers at the time. I had attended a Yale IT meeting where the migration of YSN IT to central Yale IT was the focus and asked when PHP programs would be allowed to reside on a Yale IT server. I had heard rumors that PHP would soon be allowed. Essentially, I was told it was not going to be in the near future and thus would be of no value to me when it occurred.

Fortunately, as it turned out, Yale also runs a special Yale research server where anything that does not violate the law and decency can be uploaded and run, but mostly faculty research projects were uploaded to this server. Why no one had previously informed me about this option, I do not know. The server allowed PHP and was monitored, but it was not maintained by the Yale research server staff. Thus, I had OSU IT graduate students maintaining the YHS survey. The YHS website was only down for a brief period when a coding error was accidently introduced while updating a line of code; it was rectified in less than an hour.

Thus, with the YHS Web survey instrument hosted on the Yale research server, I thought we were ready to launch the YHS study onto the wider Web. However, a few more obstacles arose causing more delay.

### The Security Design Review

One item that created a high degree of frustration and delay was the necessity of conducting a security design review (SDR) to ensure we were compliant with the Health Insurance Portability and Accountability Act (HIPPA) data protection regulations to safeguard personal health information of study participants. The YHS survey instrument was designed to be anonymous to facilitate participation and candid responses. However, my previous face-to-face interviews with ACS patients demonstrated that YHS participants may need an opportunity to return to their survey to make changes, to complete the survey if they ran out of time or were interrupted, or if after they completed the survey forgotten information came to mind. A way of allowing them to return to their survey needed to be designed and built. Because the survey contained medical history data, the log-in procedure, personal ID password system, and servers needed to be HIPPA compliant. If Yale IT had rewritten the survey, HIPPA compliance would have been written into the survey program, but because it was coming from an outside source, an SDR was needed, especially since the OSU IT graduate students writing the survey were not fully aware of HIPPA because they were not on the medical side of campus.

Yale IT charged a princely sum for the SDR. I did not feel that I should bear the cost of this review because the shift to central Yale IT was not anticipated or budgeted for. After intervention by the dean of YSN, I received assistance with the review without a fee. Thus, the YHS survey could be returned to by participants, was completely anonymous, was HIPPA compliant, and all data were deidentified in the event of a server security breach. No one breached the security of the YHS website.

However, checking to see if anyone returned, by monitoring a specific number of questionnaires for a given period, no one returned as far as we could determine. However, six surveys were begun and a similar, if not identical, personal code was close by in the sequence of identification numbers with one partially completed and the other completed. These participants had begun the survey with one passcode and forgotten it, and thus they had to start anew and complete the survey. In these instances, we accepted the survey that was most completed. Yet, we do not know how many participants wanted to finish and could not get back in and so did not complete their survey. Despite the multiple options available for contacting us, no one contacted us to indicate they could not return but wanted to.

### YSN Graduate Research Assistants

Because I was anxious to get the YSN survey up and running, and Yale union hiring practices seemed an unfathomable mixture of rules and regulations, I decided to hire graduate research assistants (RAs) from the pool of graduate students at YSN and rely on available part-time RAs “floating” around YSN. Hiring YSN graduate students, I was warned, was not a good idea as the burden on the graduate entry program students (these students have a BA or BS degree and become both nurses and nurse practitioners in an accelerated program) was exceedingly high and, besides, no one would apply. Well, they were correct and also wrong. Yes, they were overburdened but still liked the idea of working part-time; they were limited to 9 hours per week (I had been accustomed at OSU to having plenty of graduate students wanting to work and being able to work 20 hours per week.) Although I was dealing with many of the issues mentioned above, I had 2 students begin to look for resources related to health websites and print ad recruitment strategies described below; this arrangement was not very productive, as students wanted to do all of their work off campus or on weekends, and I saw very little of them. Working this way did not help in the execution of the sampling strategy to recruit study participants. In general, YSN students were taking too much time off for exams, breaks, and holidays, and this was problematic in establishing a productive working rhythm.

A bit later in the study I also had 2 RAs who, as part of their PhD matriculation, were assigned to ongoing research projects. Rather than have them do something that an RA might do, they were given an independent task; one to run our YHS recruitment blog postings on heart disease and healthy behavioral changes, and the other to expand our outreach to potential African American participants.

### Beginning to Collect Data

In the social and behavioral sciences, we like to have as much control over our sampling frame as possible to make certain that we are sampling the correct population and are not introducing systematic biases in data collection. To gain some semblance of control and to assess YHS’s representativeness, we turned to the National Health and Nutrition Examination Survey (NHANES) to estimate the size of our potential target population. We had, in 2004, a target population of 7.8 million individuals who had previously experienced an ACS event [8]. On the basis of the NHANES, we constructed sampling quotas stratified by sex, age, and race, white and African American, based on the prevalence of ACS events derived from the NHANES 1999-2000 [9] and 2001-2002 [10] studies. We would try to match the quotas in each category, thus providing a gauge of the representativeness of our sample. At the time the grant was prepared, the NIH had a policy of making certain researchers include African Americans, women, and older participants. The quotas developed around these three characteristics made us monitor our emerging sample and make modifications to our planned recruiting techniques as we progressed.

### Oversampling African American Participants

My experience with the two prior ACS studies, described above, and the known difficulty in recruiting African American research participants [11], made me over sample African American by a factor of 2 to be certain that African Americans were adequately represented. We proposed recruiting 386 African American participants distributed by age and sex. We failed abysmally to recruit African American participants, with only 33 (8.5%) of the planned 386 being recruited. Of the 33 participants who completed the survey, only 27 provided usable time information and will be included in the analyses. I did not anticipate such a low level of African American participation. It is obvious that the digital divide has not been overcome as the literature [12] had led me to believe. I will detail our efforts to oversample African American participants as we proceed.

## Recruitment Techniques

### The Five Techniques

To fulfill the sampling quotas, we needed to attract potential participants who had experienced an ACS event. I did not believe that it should be too hard, as we only needed 2314 participants out of 7.8 million individuals in the United States who had experienced an ACS event. To reach the eyes and fingers of potential study participants, we devised five broad techniques to place ads: (1) on public social media websites; (2) on conventional public bulletin boards in public libraries, senior citizen centers, cardiac rehabilitation centers, and in African American churches; (3) in chat rooms, public forums, or electronic bulletin boards; (4) in the form of viral email marketing; and (5) in traditional print publications, for example, American Association of Retired Persons (AARP) or college alumni magazines. Ultimately, we spent most of our time and resources recruiting with paid Web ads on social media and contacting libraries and senior citizen centers to post our flyers on their community bulletin boards. Along the way, we devised additional techniques as we built upon the success of one method, while curtailing the emphasis placed on less successful ones, described below. The basic strategies were continually evolving and emerging over the 2 years, 4 months, and 16 days of actual data collection, excluding partial blackout periods described below. Each time we altered a technique or began a new one, we submitted an institutional review board (IRB) amendment; all were approved in a timely manner.

### The Two Dominant Recruitment Techniques

#### Social Media Ads

We began our recruitment by running Internet ads on Facebook as well as Google and its family of websites. We added AOL and Yahoo or Bing a bit later, partially because they are known for being more popular among older potential participants, and we wanted to see if we could increase male participants who were not initially showing up to fill sampling quotas.

#### Bidding on YHS Ad Placement

Placing ads on social media is not a simple task. Knowing, as we all do, that we rarely go past the first page of our Google or Yahoo searches, we had to bid how much we were willing to pay for each click to the YHS website. The more you are willing to pay per click, the more likely your ad will appear on the first page of someone’s search for information about the heart or heart disease. This also meant we had to select keywords and phrases that were likely to be searched by potential participants who had experienced an ACS event, for example, heart symptoms, I have chest pain, angina symptoms, heart attack women, or heartburn heart attack, to name a few of the 878 terms we used, including misspelled terms like “heart attach.” So, when someone searched using one of our keywords, our ads would appear on the first page of their results, provided we bid high enough.

Having a budget for a given length of time, it was difficult to know how much to bid at first. It took time to overcome some frugal tendencies to bid against more well-funded pharmaceutical companies, purveyors of questionable heart disease nostrums, and university and for-profit health care systems. All Web providers allowed us to target potential participants by age and sex. Both Google and Facebook had the highest cost per click (CPC) ranging from US 50 cents to US $5 or more, whereas AOL and Yahoo or Bing were in the cents per click range. Additionally, we had to set weekly spend limits for each provider, whether US $50 or US $275 as we did near the end of our funding.

On average, we paid US 0.89 cents per click to the YHS website, meaning that each time someone clicked one of our ads and were taken to the YHS Web page, we paid the vendor or Web page owner our bid price. You never knew whether it was money well spent or not, but since we had so many clicks and so few participants, it is easy to suggest that it was not very efficient. It was also troubling to think of the possibility that students writing class papers about the heart or heart disease clicked on the site hoping to gain information, or that the ads never really ran because we could not have possibly monitored their display. However, one inquiry to Google about this possibility quickly brought back an image of our ad on a Web page as verification of ads’ having been run.

Nonetheless, in bidding on the four social media hosts, we did not know whether the ads were being looked at by potential participants who were suspicious that they might be having an ACS event, those who were looking to see if they were at risk for a heart attack, someone wanting to prevent a heart attack, someone who wanted to know if someone they knew might be at risk or having an ACS event, or those who were searching post an ACS event and wanted to understand what had happened to them medically. In addition, some individuals may have been looking at the YHS website to help their friends, as in a Strauss “information provider” [13], which may have been the reason why we had so many individuals who registered on the YHS site but failed to progress beyond the first question; more on this point will be discussed below. In a compassionate manner, these information providers may have been trying to understand what a family member or friend may have gone through during an ACS event.

Although we wanted to avoid having our ads run on websites selling commercial products and questionable health nostrums, we, in fact, had little control over where our ads were placed. However, judging from the sites that our YHS ads were clicked from, there was a wide variety of websites where our ads appeared; this was not a problem for Facebook as all ads were run on their site alone. A website from which a potential participant came was not an absolute indication of where they saw our ad, as they could have noted down our website URL from a site on which they happen to have been when they decided to check out the YHS site.

We did not use real-time bidding services to tweak our bidding; these had become more prominent after the study was designed. However, we did respond to contacts from the 4 social media vendors to have them evaluate our campaigns, and I generally found it beneficial but ultimately more expensive, as their specialists enviably recommended ways to increase our limit on spending or our CPC bid. They were most helpful in assessing our selection of keywords and how to target our ads to African Americans, men, and older potential participants. They also showed us newly added social media features and how to use them. Additionally, we paid extra, usually between US $5 and US $30, to promote our YHS Facebook page when our posts began trending upward.

Using the hit rate to the YHS site, our budget, the success of keywords and ads in securing Web page hits, adjusting our bids to be on the first page—someone new can come in at any moment and bid really high—and, of course, the need to monitor all 4 social media vendors, the recruitment by the Web is a very time-intensive activity.

Ultimately, in a very real sense, we were living and dying on the basis of our ability to run ads on the social media sites.

#### Public Libraries and Later Senior Citizen and Cardiac Rehabilitation Centers

Our second most intensive recruitment effort, in terms of personnel hours, was soliciting the cooperation of public libraries across the United States to post our advertising flyers on their community bulletin boards. A bit later, we began targeting senior citizen and cardiac rehabilitation centers to also post our flyers.

#### The Libraries

We contacted every public library in the United States that was listed on Public Libraries website [14] and for which we could locate an email address. We also used, when available, state lists of public libraries, as well as state listings for senior centers. There was considerable variability in the openness of access to contact information for library directors. The range of availability was from a picture of the library director on the library’s Web page with an email address directly below to zero information on how to contact the library electronically, aside from a general telephone number for the library. If the city was relatively large and with multiple library branches, we directly contacted the central director rather than contacting each branch individually because in all probability branch directors would contact the central administrative office, and we did not wish to appear as if we wanted to circumvent them. Thus, it was an all-or-nothing approach in larger communities, for example, Los Angeles, California. Central directors frequently let us know whether they were sending out our materials to branch libraries because we had established a relationship with them, as additional materials were usually required for their approval.

#### The Senior Centers

In terms of senior centers, locating a list of centers for each state was the main task. In both public libraries and senior centers, no simple criteria were established in terms of how long to search on the Web to find an email contact. In the extreme, we would look for minutes of city council or governing board meetings because emails of city board members and significant directors would frequently be listed as part of the meeting minutes; websites of city managers would also have emails of directors, whereas the library website itself would not. We tended not to telephone libraries, as we initially thought it would not be efficient. We did relent, however, and telephoned using a standard script to request the name and email address of the director. Our request was never refused, and in very small libraries, the director was usually manning the telephone. At times we felt like quasi-spammers sending out hundreds of emails and stalkers when we went as far as checking on LinkedIn and Facebook for an email address.

Alternatively, we also used the “Contact Us” box for the library circulation desk or reference department and asked that our information be forwarded to the director. In these instances, we sent a modified cover letter with links to our download website, discussed below, because it was not possible to send our attachments through the Contact Us box. Reaching out to the city manager was also an effective way of contacting a small library or senior center director, especially those open for very limited hours

In many instances, contacting librarians, because we could not find an email address, proved beneficial as they made useful suggestions for advertising the study. The first time this occurred was when a librarian in California suggested that her patrons really liked bookmarks. So, we ultimately designed, printed, and mailed out hundreds of them each week to not only libraries but senior centers, cardiac rehabilitation centers, churches, and professional health care provider meetings willing to distribute them.

An additional suggestion by a librarian was that we translate our bulletin board flyer into Spanish. This was particularly useful in the West and Southwest. Due to the study design, and later, cost and time, we did not have a strategy to target Hispanic participants, and we did not have the YHS survey translated into Spanish.

We believe some libraries and senior centers opened our emails, went to the printer, and immediately posted our flyer, whereas others took considerable time processing our request through a committee or person who oversaw access to the public bulletin board. In cardiac rehabilitation offices, which were usually connected to hospitals, we also had to overcome the hurdle of an IRB or research committee review and approval process. In these instances, when requested, we responded with copies of Research Aims and Methods sections of our grant proposal and of our Yale IRB approval letter. In all instances where we were appraised of the approval process, we received approval. Also, each library, rehabilitation center, and other posting organization had its own policy governing the length of time an announcement could remain posted. Thus, another important thing you lose is control over the exposure and sustaining power or half-life of your advertising efforts.

#### Multiple Web Pages for YHS

Originally, we sent libraries a cover letter soliciting their cooperation, including a PDF file of our IRB approval letter, a newsletter paragraph for use on their website or newsletter, a flyer with and without pull tabs they could print, and 2 box ads files for website posting. In some instances, these items were cumulatively too large to pass through email filters, and we subsequently reduced the size of our email package. We eventually developed an additional YHS download website from which librarians and others could download and print our materials or request that we send them our materials. This download site also meets the needs of a commercial email advertiser, discussed below, because they would not send out attachments in their email “blast,” as they called their mass emailing. This download site served us well, as we had 701 download requests and mailed out hundreds of packages of bookmarks, posters, and flyers across the United States.

To avoid burdening librarians, we only asked for their email system to tell us that our email was received and not whether it was opened. Unfortunately, we received very few acknowledgments indicating that they would cooperate, but when they did, they did so enthusiastically noting that an ACS event had touched them personally or a family member. It was not unusual to be scrolling through a library’ website or for a YHS team member to drop into a library while travelling and find our flyer or poster being displayed. After solicitations to libraries were ended, we began receiving notification that our email had been discarded without being opened; this reflected email systems clearing out unopened emails!

#### Chat Rooms and Changes in the Web Environment

In the original design of the YHS, I had proposed using chat rooms to disseminate information about the study and to post our YHS website URL link. During my initial exploration of these sites, in preparation for the grant proposal, all comers were welcome; information about products, services, and studies were relatively ubiquitous; and monitoring or restrictions were minimal. These websites had not yet been fully monetized and advertising seemed plentiful. By the time we returned to these sites, relatively strict rules and policies were in place. When we were ready to launch the YHS website and begin our advertising, we first read the prevalent “use agreement” of any site that appeared as a possible venue for YHS information and ads. Almost all websites that would have been useful now prohibited the “recruitment of research subjects.” We did find a suite of sites, Daily Strength [15], which has numerous support groups covering a wide variety of chronic and acute health conditions. I reviewed their use agreement statement very carefully and noted that they did not prohibit the recruitment of research subjects, or so I thought. I uploaded one of our paragraphs describing the YHS and the URL of the YHS website to several support or self-help groups whose members might have had or be at risk for an ACS event; for example, I posted on their hypertension, diabetes, heart attack, cardiac arrest, women’s heart health, and high cholesterol groups. We had a very good response, especially from women who could perhaps be described as having had a “microischemic” ACS event. One women had immediately signed up to participate in the YHS and then encouraged others participate; she was obviously a sociometric leader of the group of relatively young women. To facilitate participation, I responded to comments from group members who found the study useful and thanked them for participating.

The rapidly changing nature of Web information and the relatively short half-life of that information were driven home by the fact that engagement with the YHS lasted until another “hot” topic was posted. At that point, the presence of the YHS would be lost without a thread of comment. It was in going back to the support groups to remind them of the YHS that I was “caught” and told that research recruitment was not allowed. Consequently, all references to the YHS were removed from the entire suite of sites instantaneously. I pleaded my case indicating that no research recruitment prohibition was stated, but no response was forthcoming. However, I was subsequently informed that we could become a sponsor of the website and advertise with them as a site sponsor. I prepared an application to determine the cost of such sponsorship but never received a response. By this time, other pressing matters were confronting us, and we did not follow up with them.

Finally, early on we looked into running ads on WebMD, a very popular medical information website. At the time, they quoted US $20,000 to run our ads for 1 month. After some discussion, they were willing to sell us a 2-week period for just US $10,000. At the time, this seemed expensive and so we declined. I do not know just how much more successful we could have been had we gone with them for a 2-week period, but it would have been almost 10% of our original advertising budget.

#### Viral Marketing of the YHS

As part of the strategy to bring potential participants to the YHS site, we built in a viral marketing option for individuals who were either curious for someone they knew had experienced an ACS event or who happened upon the YHS site. What they needed to do was click on our “Visiting for Someone Else” button, and they were provided with a form to enter their first name, the first name of their friend, and their friend’s email address. An invitation was sent to their friend describing the study and the fact that their friend was responsible for us sending the email. Email addresses were not collected, and a statement to that effect was present in the form and in the received email. A total of 163 invitations were sent out using this method. In general, I would not describe the method as effective, and we do not know how many of our final participants made a decision to participate based on receiving our email. We did not advertise the fact that we had this method available for individuals to send invitations to potential participants. We will assume that anyone finding us also had the option to send the YHS URL on their own to a potential participant.

#### Print Ads and Media Outlets

We never published a print ad, as I had proposed. We found that it was more expensive than I had originally estimated, and there was considerable lag time from ad submission to publication, usually several months. In addition, a part of the expense that I did not anticipate was ad design. Newspaper ads were ultimately decided against because they were too local, and larger markets were too expensive.

At one point, we decided to place ads in university alumni magazines, especially those with a large alumni base, such as Michigan or Ohio State. But as we were not having problems recruiting educated participants, and if anything we were slightly biased in this regard, so we abandoned this plan as well. Instead, we were able to go onto the Facebook pages and Twitter feeds of various universities and alumni groups and friended and tweeted them our YHS link with a brief study description; we also friended and retweeted information from nationally well-known heart institutes and programs, for example, the Cleveland Clinic, Cleveland, Ohio, and the Texas Heart Institute, Houston, Texas. The overall assessment was that we probably received more interest from these Web-based efforts than print ads.

We did, nonetheless, have print coverage from various sources. The Office of Public Affairs at Yale University and the YSN both published studies covering the YHS. One of our RAs who had worked in the New York City media market had tried to obtain a spot for us on the Dr Oz and Oprah shows but was never able to obtain a firm commitment. However, she was also an occasional contributor to the *Huffington Post* and did publish a marathon of heart health–related editorial columns during Heart Month of 2012. We experienced an increase in website hits during this period.

We also focused on media outlets to cover the YHS as a news item. In this effort, I did two radio interviews, one email interview with questions provided for answering, and one newspaper interview with the *Wall Street Journal* (WSJ). After the WSJ study about ACS in general, we received a substantial bump in traffic to the YHS website. The WSJ was the most national coverage we received, and it was evident that advertising nationally is probably an efficient means of enhancing website traffic if funds are available.

#### New, Modified, and Emergent Recruiting Techniques

##### Email “Blasts” to African American Pastors

As noted above, the oversampling of African Americans was part of the study design, yet it yielded only 33 African American participants. To facilitate the oversampling of African American participants, we used three distinctive techniques. The first was a two-time email “blasts” by a proprietary emailing company to solicit the cooperation of African American pastors in communities throughout the United States. The company we used originally told us that their list included the names and email address of 1700 African American pastors, when in fact they provided only 1200 pastor addresses. We complained and were given an additional blast. Neither blast produced tangible evidence of success or increased spike in African American participation. However, we did receive two telephone calls that our materials would be disseminated to parishioners. Before this effort, I was to consult with a panel of African American pastors affiliated with the Yale Center for Clinical Investigations, a center that facilitates research with difficult-to-reach populations, but the meeting was canceled. However, I was able to consult with one pastor in the group who suggested my solicitation letter should be more compelling in terms of why a pastor should assist us. Apparently, I did not make the letter compelling enough.

#### Targeting Communities With High African American Populations

In addition to email blasts, we obtained from the US Census Bureau a listing of the 66 metropolitan areas and cities in the United States that reported 23.7% or more of the population being African American residents; for example, Detroit, Michigan; Memphis, Tennessee; and Jacksonville, Mississippi [16]. After our email solicitation techniques were finalized, we immediately targeted the public libraries and senior citizen centers in these 66 areas and cities.

#### The African American–Focused Health Website and Traditionally Black Colleges and Universities

Lastly, we contacted the most popular African American health bloggers and health websites to place ads with them. This was not successful both in terms of their response to our inquiries or the number of times we found our Web ads on their websites. African American health websites were notable for their lack of a direct focus on the prevalent health needs of African Americans and instead focus on physical appearance and lifestyle matters. It was rare to find among African American–focused sites information regarding health risks and behavior modification in prevention and treatment of high blood pressure (HBP), diabetes, or heart disease. I would assess their health information as “soft” at best.

As noted above, we “Liked” on Facebook and tweeted heart health information on university alumni sites, and we did the same for historically and predominantly black colleges and universities and their alumni groups.

#### Young Take Older to YHS Site

We approached youth-oriented websites to run our ads that encouraged more computer-literate high school students and young adults to take less computer-savvy family members, who had experienced an ACS, to the YHS website and assist them in completing the survey. I do not know how comfortable this was for older adults, but we assumed that for those who participated it was not an issue and this effort may have increased participation among the elderly. In addition, while working with state 4-H groups and the national organization of school librarians, we asked for their assistance in recruiting the young by displaying our posters. As an inducement for younger individuals, we provided a certificate for 1 hour of community service from YSN. We do not have complete records on this program to say how successful it was, but we did send out at least 100 certificates.

#### Walk With a Doc

We also spent a very nominal amount, US $50, for a national newsletter ad with Walk with a Doc (WWAD) [17]. WWAD is a national organization that promotes healthy physical activity among at-risk and chronically ill individuals. They sponsor Saturday morning walks where interested parties can spend a couple of hours walking and talking with a physician who is willing to share his advice on health and well-being. Again, I have no idea how effective this effort was.

#### Avoid Your Own Backyard

In general, I tried to avoid recruiting in the environs of New Haven, Connecticut. I wanted to obtain a nationally representative sample of participants and to fill our sampling quotas. Despite the fact that RAs wanted to have more control over the sampling and wished to reach out to locally available participants, I resisted the temptation to pick the low hanging fruit. For this reason, I also resisted recruiting in the New England area, until we had been to other regions of the county with our library and senior center flyers.

#### Reward for Participation

As an appreciative gesture for participation, we maintained a Facebook page, Twitter feed, and YHS blog, providing a variety of health information links and studies related to heart health, heart disease prevention, diet, exercise, and other news items that might be of interest to study participants. We developed these information sources because we could not maintain anonymity and easily provide participation incentives. Also, these three social media platforms were also used to recruit participants to our study site, as we would periodically ask followers of these social media outlets to participate if they had an ACS event or knew someone who had had an ACS event; again a viral-type effort. Despite the study having ended some time ago, I still maintain the Facebook page and still post 2 to 4 items per week, depending on the number of people reached by the post—if it is something that is very popular at the moment and/or something that the individual can do to modify their heart health or risk, I may run it a full 7 days, but rarely does a Facebook posting have 7 day legs.

#### The Comments and “Other” Response Options

Whereas participation in the YHS was anonymous, some participants chose to provide us their email address in the event we needed more information or, surprisingly, if we wished to know the name of the hospital where they were treated to obtain their medical records. This offering was bittersweet, considering my effort to protect their anonymity and the SDR difficulties noted earlier. In the YHS, 271 participants left additional comments. In the Bethesda study, either my coinvestigator or I, or nurses in the Columbus study, had written detailed narratives for each study participant [18]. These were useful in understanding the behaviors and reasons for patient’s actions as well as for developing the YHS survey instrument. The Comment section in the YHS survey and the “Other” response option in almost all questions provided useful information to supplement or clarify time calculations and event sequences.

## How Did We Do in Recruitment, Final Yield of Participants, and Reasons for Noncompletion

### Web Ad Stats

From June 1, 2011 to October 31, 2013, using Facebook, Google, AOL and Yahoo or Bing, and their families of websites, we displayed 279,834,651 impressions of YHS ads that generated, we believe, the vast majority of the 124,795 clicks to the YHS website since November 28, 2011, when we began tracking website hits; we tracked YHS Web activity for 1 year, 11 months, and 4 days or 704 days. During this tracked time, we averaged 177.3 hits per day to the YHS site. Over the total course of recruitment, which spanned 884 days, or 2 years and 5 months, we averaged 2.7 sign-ups per day to participate, meaning potential participants clicked to the YHS website, clicked on the button indicating they wanted to participate after reading our study description, consented to participate by clicking on an “Agree” radio button, read an instruction page detailing variations in page format and how to get definitions of medical terms, created a personal identification code, and progressed to the first question of the YHS survey. It was interesting that not unlike any Web vendor on the Internet, the YHS study had about the same ratio of clicks to the site for each completed survey as a vendor selling items on the Web.

There was one major 6-month interruption in data collection because of an administrative matter, discussed below, when no Web ads were run, and fewer library or senior center solicitations were sent out. During our two fully funded periods, we signed up 4.5 participants per day, in contrast to our unfunded period when sign-ups dwindled to 0.53 per day. There was a natural nonfunded period at the end of the study; because the YHS still had a presence in libraries, senior centers, rehabilitation clinics, and on blogger websites, the YHS website remained up for 184 days after funding had ceased to support RAs, printing, and Web ads. The IRB approval remained effective during this period. During this 184-day period, 81 additional participants or 0.44 participants signed up per day. What was curious was that during the periods when we posted no Web ads, the quality of the data actually improved, meaning that there were far fewer noncompleters than during fully funded advertising periods. We speculate that these participants were highly motivated and perhaps put off their actual participation until a later time, whereas during funded advertising periods, we attracted more persons just exploring our website.

What was obvious to us was that the most consistently productive recruitment method out of those we used was ads posted on Web on the four social media providers, Facebook, Google, AOL, and Yahoo or Bing. We spent US $112,303.98 in advertising on ads placed on these four providers. In constructing our social media ads, we found that the most effective ads were those that appealed to the “altruistic sense” of a potential participant. Our most successful ads, determined by how many clicks they received and the click-through rate—seeing the ad and then clicking through to the study website—had some variant of the following: title: Yale Heart Attack Study; ad text: Help Others by Sharing Your Heart Attack Experiences; and a radio button to click to the YHS website. We tried many ads, even seasonal ones, but none worked better than those with an altruistic appeal.

This leads us to consider how many individuals actually completed the YHS survey and how many usable surveys were actually obtained. Of the 2381 who signed up on the YHS site, 1886 progressed beyond the sign-up and first question, “Before your most recent heart attack, had a doctor ever told you that you had heart problems?,” and 1208 completed the survey by sharing their demographic characteristics at the end of the survey, which was our gold standard for completion. More importantly, 1154 provided sufficient information regarding the primary dependent variable of total time from acute symptom onset to ED arrival. The first cut of the 2381 participants was made at the first question that needed to be answered to progress in the survey, and thereafter, participants dropped out until the end of the questionnaire. Total time computations presented issues where participants would complete all of the survey but leave most time designations blank; participants who only provided certain time information but not enough to compute total time; and participants’ whose time information did not allow for an accurate computation or was confusing with regard to the time of the day or the actual number of hours or days. The computation of total time was supplemented in 218 cases from information provided in the “Other” responses and “Comment” section. When none of these information supplements were available and a coherent timeline of events could not be constructed, participants were removed from the analysis.

### Nine Reasons for YHS Survey Noncompletion

So why did we have a completion rate of just 48%, when it took quite a bit of diligence to sign up, which in turn should have made a potential participant feel committed to finish? There are several reasons why potential participants may have seemed committed to participating but failed to advance to the end of the survey.

First, it is possible that despite the fact that we say we are interested in people’s heart attack care-seeking behavior, some people may have thought we were a study of the “heart” that might have some useful information for them about heart health, research findings, and the like.

Second, they are just curious about what might be behind the wall and registered only to find that the first question was about whether they have heart disease and stopped, although to get that far they must have seen what we were about from the study description, consent form, and navigation instructions.

Third, some people registered and decided they would complete the survey later only to forget their initial personal registration code. So, they reregistered, leaving us with one incomplete survey, as described above, and just did not feel motivated enough to do it all over again.

Fourth, they felt that being asked whether they had a heart disease was not a good way to begin a survey, and they stopped immediately because they found the question threatening, and/or the survey aroused feelings of PTSD that were still prevalent from the ACS event in question. In covering identical material in face-to-face interviews, I realized that at times it was difficult for patients to answer questions about a life-threatening experience; it was as if they were reliving it all over again.

Fifth, there may have been survey fatigue. It was a long survey for some participants, although the self-tailoring design did reduce fatigue somewhat. Furthermore, it was almost halfway through the YHS survey, after the medical history and ACS warning symptom sections, before they arrived at questions about the ACS event itself.

Sixth, although we carefully designed the YHS instrument to download and upload quickly, computers and connections may not have been fast or reliable enough to sustain a reasonable pace of progression, and participants may have become impatient or disconnected.

Seventh, in terms of care-seeking time entries, participants may have been unable to recall times for an event that may have occurred sometime before; some participants told us they became unconscious and were not aware of time, although there were some indications that in these circumstances participants had consulted with someone else to complete the survey. If no total time calculation could be discerned, participants were not included in the analyses.

Eighth, some individuals attracted to the YHS might not have been aware of what it means to participate in a research survey study and were not prepared for the experience in terms of the demands to recall a potentially life-threatening event, to read numerous questions, and to select among many answer options and checkboxes.

Finally, there may have been an “easy come, easy go” mentality. That is, potential participants found us, but they had no commitment to us. After all, we did not personally reach out to them, and they just as easily left us at the slightest distraction, interruption, discomfort, time pressure, or whatever deterrent broke their bond of connection to the screen reaching out to take their ACS experiences, even though they might have felt that they were helping others. The type of engagement that Web-based research creates maybe just too ephemeral to sustain without the existence of a personal bond between a participant and an interviewer. Web-based research efforts need to be aware of that balance between the comfortable security of anonymity and our investment in creating a bond strong enough to sustain them through to survey completion.

## Distractions, Impediments, Delays, and Unsuccessful Recruiting Techniques

As already noted, there were many impediments to launching and to completing the study on time. Here are the most significant contributing factors for delays, some distractions that were unexpected, and some seemingly useful recruiting techniques that, in fact, failed.

### The “No Cost Extensions”

Due to the many impediments and delays described above, it was necessary to apply for no-cost extensions (NCE) from NIH. This meant that my projected time to complete the study had run out. We could do the study in 3 years, but at the end of 3 years, I was not finished collecting data; however, I still had funds to continue the study. So, one asks for an NCE to continue working. Rumor has it, and it was confirmed, that the first extension is easy, but the second extension is much more difficult. The rumors are true; the first one was merely a signature on a form. However, the second NCE required undertaking an extensive reapplication, and there was a full 2.5-month delay until it was finally approved. Interestingly, no one at YSN or Yale’s Grants and Contracts office would approach NIH about the lost 2.5 months that were subtracted. Apparently, time being cut is just part of the process, especially on a second NCE request. Additionally, even more delay was experienced when I asked for the unprecedented third NCE.

The first NCE had no effect on data collection because it occurred before launching the YHS website. To some extent, the second NCE did not cause any disturbance in data collection either. Since there were sufficient funds remaining and the prospect of a second NCE was probable, we were lent the funds by Yale to continue data collection as we had just begun in June of 2011, and the approval was given in July of 2011.

We had built up full momentum by the end of the study’s second NCE period. We were attracting potential participants to the YSN website, we had established a network of reciprocating websites and organizations whose focus was cardiovascular disease and heart health, we had a routine getting out our materials to libraries and senior centers, our Web ads were getting above-average hits for a noncommercial entity, and our efforts to reach out to other media, essentially health bloggers and organizations with target populations of interest, were successful and expanding.

As it is rare to obtain a third NCE, YSN was not going to front the YHS despite the remaining funds, and as NIH was going to lay a heavy penalty by delaying approval of the third NCE, we essentially closed for 6 months. Personnel were let go and one assistant was retained from funds from internal sources for 2.5 months, and I took over the outreach to senior centers. Emphasis on senior centers had an unanticipated benefit; we were in need of older participants, and during this 6-month period, we were able to raise the average age of study participants by 1 year.

As we did not have funding during the penalty period, I could not advertise for or interview an RA in anticipation of an approved third NCE application; funding needed to be in hand. The absence of Web ads during the NCE application period severely diminished the number of clicks to the YHS site. Enrollment continued but drastically reduced from 4.5 per day with funding to 0.53 without. I assume that these later enrollments were in response to flyers that were still posted in libraries, senior centers, and other places; Web-based banners and box ads; and from the followers on Facebook, Twitter, and the YHS blog. During the unfunded periods, I continued to respond to questions from various people who wanted information, responding to Facebook postings, and screening YHS blog comments that were mostly from companies trying to gain publicity by posting a comment, which usually did not have anything to do with the YHS blog content. We discontinued sending out requested materials because of a lack of funds for printing and postage.

After approval, when the third NCE finally came through, it took 2 weeks to regain prior enrollment rates. However, if there was any benefit from being unfunded, it derived from the ads appearing new to both new information seekers and to those who were previously ad fatigued, who may have seen ads anew when we started up again. Web providers must have a priority for taking on new advertisers, and we were placed at the end of the queue and had to work our way back by bidding above going rates to place our ads on the first Web search page.

### I Want to Join Your Study

I do not know if this is a common occurrence in Web-based studies, but 3 physicians wanted to join the study and become an integral part of the team after coming across our YHS ads. Their entrance ticket was to be access to their population of patients who had experienced an ACS event. One wanted to be included in publications, and another was much more explicit in this request, needing publications for an upcoming tenure review. The more subtle approach merely mentioned collaboration and merging of datasets. My response ranged from “this is impossible as I already had a complete team, including a cardiology consultant,” to “I can set up an alternative website to collect data and you can send your patients to the site, but I have no funds for your personnel to cull names from your medical records.” Needless to say, no one followed up on my offer of another website and the use of their personnel to generate the list of potential participants.

### Illness of a Research Assistant and Unions

The restrictions of union hiring were evident again when a key RA required emergency surgery shortly after being hired. She needed to recover for several weeks, and the process of hiring a new RA would have been almost as long as the projected recovery. Thus, for several weeks we were without any canvassing of public libraries. However, on the up side, the assistant became highly motivated and extremely productive upon returning; perhaps they were not feeling well during the period before their surgery. As a result of this increased productivity, we were able to meet our goal of sending to every public library in the country, for which we could locate an email address, our solicitation package.

### The Evolving Internet Environment

The nature of Web advertising changed over the course of the study and when the YHS website was taken down. More tracking techniques became available; for example, if one currently entered on Google or AOL the term heart attack, for the next month everywhere you surfed on the Web, our ad would have appeared encouraging and soliciting participation in the YHS. We did not have the resources to pursue tracking techniques early on when they were developing. I do not know if currently people find these tracking techniques annoying enough to discourage participation in a Web study.

### Unsuccessful Techniques

We tried contacting restaurant associations in each state to see if they would send out a newsletter item about the YHS and whether members would be willing to post our materials on the bulletin boards one commonly sees filled with local tradesperson’s business cards and flyers for pancake suppers. This did not prove to be effective as hardly anyone returned our calls and, of those who did, no one agreed to participate.

### Modifying the YHS Instrument

Suggestions to modify the survey were met with much more consideration of scientific design and integrity; for example, adopted participants in closed adoptions could not answer whether they had heart disease in their family or if anyone had heart disease before the age of 50 years, which is a significant risk factor. Since genetics and lifestyle are both a part of risk, it is difficult to know what was ultimately more important a factor, family of orientation or birth family. As a compromise, a “Do not know” was added as an option. Also, one person wrote indicating they could not progress in the survey. I went to their survey and tried to determine why they were having the problem. As it happened, 2 questions back they may have entered an incorrect response by mistake, thus limiting access to where they thought they should be going given how they had answered prior questions; they had great insight into the logic of the self-tailoring algorithm. I wrote the participant with the solution but did not check to see if they followed up, as I wanted to maintain their anonymity as best I could.

No one in the Comments section commented on our advertising or suggested alternative approaches. Aside from 3 comments regarding questions in the survey, we received no criticism about the survey except that some sections seemed repetitious and they were as we wanted to know, for example, what did lay others advised in each care-seeking phase and we also wanted to know what self-treatments were used in all phases except the travel phase. In both cases, among others, all questions were identical, and if one experienced all care-seeking phases, the repetition was more than obvious. As noted above, fatigue may have set in, and repetition of questions may have encouraged discontinuation.

### Spammers Effect on Web Studies

Cyber-war defenses to keep professional spammers from capturing names and email addresses from Web pages and Web page directories placed many barriers in our path. These efforts ranged from not posting vital emails to not allowing us to copy from websites vital information for contacting library and senior center directors. Thus, as noted, we sometimes relied on city managers among others as sources of information. As the cyber-wars have advanced, this type of study, with a national focus, may be more difficult to mount without major assistance from professional Web marketing organizations. However, noting that California had the most barriers to email access than any other region of the nation, it would seemingly be difficult to find one approach or technique that could sufficiently overcome a myriad of defensive barriers.

### Personal Computers and Laptops, But No Mobile Phones

At the time I entered the Web ad world, ads on a mobile phone were just beginning to appear. I accessed our study website on a mobile phone and decided that the tiny image of our survey was far too small for our target audience to complete on a mobile phone. So, we only placed our ads on desktop and iPad-type devices. As mobile phone screens have increased in size and clarity, it may now be more appropriate to consider advertising on such devices and to modify a questionnaire to fit on mobile phone screens.

### The End Was Near

As we neared the end of our final budgeted period, we calculated a spend rate that would provide one final bump in potential sign-ups. This was effective as website hits increased. However, the hits were not by persons likely to participate, and the noncompletion rate was higher than when no funding was available and potential participants were driven by flyers in libraries, senior centers, and rehabilitation centers. So, higher nonparticipant hits or noncompleters may indicate that broad advertising is not the most economical means of reaching a target audience. Obviously, access to lists of discharged ACS patients would be the most effective targeting. But such a focused list again has its own regional, hospital, medical practice, and EMS biases and limitations.

## Lessons and Assessments and Final Comments

### Are Web Surveys Worth the Cost?

In the prior ACS study [18] conducted in Columbus, Ohio, using 6 nurse interviewers, we obtained 1102 analyzable interviews from 1317 eligible hospitalized patients who were recruited to participate in an interview covering their ACS care-seeking experiences; 11 refused and the remainder died before the interview. Which is the better technique for studying ACS events, face-to-face interviews or the Web? It is very difficult to say, given the nearly equal yield of study participants—1154 in the YHS. However, the quality of the data may have been a bit better in the Columbus study because some questions in the YHS were not answered or left blank where the type of question asked should have yielded a response—not an unusual occurrence in Web-based studies [19]. It is much easier to disregard a Web-based question than resist an interviewer armed with alternative ways to ask a question and a look of disappointment when the participant does not answer. On the other hand, we do not know how much the desirability factor affected results in the Bethesda and Columbus studies. In terms of monetary costs and adjusting for inflation [20], the YHS was approximately 31% less expensive than the Columbus study.

### Representativeness and Sampling Quotas

Overall, we did not achieve our sampling quotas derived from the NHANES, as described above. The sample we did collect, 1154 participants, differed from the NHANES-derived quotas, especially among elderly (>74 years), but the overall differences were not statistically significant in terms sex and age distributions from 2314 participants needed to test the ISCM.

In terms of African American representativeness, I do not believe an Internet-based study is an appropriate platform for their recruitment, despite opinions that the digital divide is closing regarding race and ethnicity [21].

### Web Surveys Are Time Consuming to Develop and Complete

As noted, conducting a Web-based study can be very time consuming, especially the development of the survey instrument and having it hosted and supported. The YHS was relatively complex and long and required more time than what most participants are willing to spend on a Web-based survey, which is about 20 min [22]. Those who finished were probably more motivated and thus may represent a biased sample of individuals who have come to have a coherent story of what happened to them and are somewhat computer knowledgeable. More importantly, individuals who do not have a coherent sense of what happened to them or those for whom the structure of the YHS survey did not resonate with their personal experience of events were lost to us when they did not finish the survey.

### Grant Transfer

I would not suggest transferring an NIH grant from one institution to another. I had the impression that the institution losing the grant was not pleased and neither was NIH with the extra administrative work. I probably should have appealed the decision of the IT director at the Department of Sociology and left the study website at OSU.

### Libraries, Senior Citizen Centers, Cardiac Rehabilitation Centers, and Email “Blasts” and Upticks

In general, noting how only a few participants signed up to take the YHS survey when we were not running Web ads, our efforts to place flyers, posters, and bookmarks in various venues was probably not very efficient in terms of recruitment and personnel and material costs. Email blasts by private vendors may be effective if one can do repeated blasts with follow-up emails and have access to the recipient’s demographics to know who is responding and who is not. But I am not certain it is cost-effective, given how much we paid for a single blast and how few responses were received. As Web-based surveys evolve, as they most certainly will, perhaps we will be able to obtain a more accurate sense of what is cost-effective recruitment.

### Final Comments

A good deal of the delays and impediments described here may be the fact of just trying to conduct a Web-based study at the time I began. I was asking for a relatively conventional, and perhaps conservative, environment of YSN to accept a research design where the buying of Web ads was especially prominent, and I was an unknown entity to Yale. Given these issues, and the perspective at YSN that the grant was given to Yale to be managed and executed by them, with me as an employee, rather than the grant being given to me and the team to be conducted at Yale with their support and assistance, I guess things did not go too badly. My logs show that I contacted 35 separate individuals to finally get the study up and running. I do not know how less encumbered the YHS should have been. I only know that my two prior efforts in Bethesda and Columbus did not seem at the time as challenging. All totaled, it took 3 extra years to collect the YHS data.

Finally, from my viewpoint, I did learn a great deal in the execution of the YHS, and now as we begin to analyze the results, I hope to contribute to the ongoing discussion of why people delay in obtaining care for ACS events that have potential for both morbid and fatal consequences over a short period.

## Abbreviations

AARP: Association of Retired Persons
ACS: acute coronary syndrome
ASO: acute symptom onset
CPC: cost per click
ED: emergency department
EMS: emergency medical system
HBP: high blood pressure
HIPPA: Health Insurance Portability and Accountability Act
IRB: institutional review board
ISCM: integrated self-regulatory care-seeking model
IT: information technology
NCE: no-cost extensions
NHANES: National Health and Nutrition Examination Survey
NHLBI: National Heart, Lung, and Blood Institute
NIH: National Institutes of Health
OSU: Ohio State University
PTSD: posttraumatic stress disorder
RA: research assistant
SAS: Statistical Analysis System
SDR: security design review
SPSS: Statistical Package for the Social Sciences
WSJ: Wall Street Journal
WWAD: Walk with a Doc
YHS: Yale Heart Study
YSN: Yale School of Nursing
